# A tRNS investigation of the sensory representation of time

**DOI:** 10.1038/s41598-018-28673-7

**Published:** 2018-07-09

**Authors:** G. Mioni, S. Grondin, D. Mapelli, F. Stablum

**Affiliations:** 1 Dipartimento di Psicologia Generale, Università di Padova, Padova, Italy; 2 École de Psychologie, Université Laval, Québec, Canada

## Abstract

The understanding of the mechanisms underlying the representation of temporal intervals in the range of milliseconds/seconds remains a complex issue. Different brain areas have been identified as critical in temporal processing. The activation of specific areas is depending on temporal range involved in the tasks and on the modalities used for marking time. Here, for the first time, transcranial random noise stimulation (tRNS) was applied over the right posterior parietal (P4) and right frontal (F4) cortex to investigate their role in intra- and intermodal temporal processing involving brief temporal intervals (<1 sec). Eighty University students performed a time bisection task involving standard durations lasting 300 ms (short) and 900 ms (long). Each empty interval to be judged was marked by two successive brief visual (V) or auditory (A) signals defining four conditions: VV, VA, AV or AA. Participants were assigned to one of these four conditions. Half of the participants received tRNS over P4 and half over F4. No effect of stimulation was observed on temporal variability (Weber ratio). However, participants that were stimulated over P4 overestimated temporal intervals in the random condition compared to the sham condition. In addition to showing an effect of tRNS on perceived duration rather than on temporal variability, the results of the present study confirm that the right posterior parietal cortex is involved in the processing of time intervals and extend this finding to several sensory modality conditions.

## Introduction

Ability to estimate the passage of time is fundamental for the good functioning of the perceptual and cognitive processes that allow the best performance in daily life activities. However, understanding the neural and cognitive mechanisms underlying time estimation remains a challenge. Even though there are no sensory organs devoted to time perception, it is possible to study temporal processing by positing that duration is processed within the sensory areas of the brain^1,2^. The idea that we time sensory signals via a single “centralised” and “amodal” clock, claimed, for instance, by the Scalar Expectancy Theory^3^, has dominated the field of temporal cognition for decades^1,4–8^. However, more recently, the universality of single clock timing mechanisms has been challenged by new theoretical positions^8,9^. The new idea is that we have multiple timing mechanisms “distributed” across brain areas or circuits, and that the engagement of each single mechanism depends on the psychophysical task, sensory modality, and lengths of time intervals^6,10,11^. Behavioural studies indicated that the ability to process temporal intervals is influenced by sensory input. Temporal discrimination thresholds are lower when time intervals are marked by auditory (A) rather than visual (V) signals^12–15^. Furthermore, when empty time intervals are marked by two brief stimuli delivered from different sensory modalities (intermodal markers), it is much more difficult to judge the time elapsed than when intervals are marked by stimuli delivered within a single modality (intramodal markers^16,17^).

It has been suggested that both modality-specific and supra-modal mechanisms underlie the estimation of temporal intervals, and evidence comes from neuroimaging^18,19^, electrophysiological recordings^16,20,21^, as well as non-invasive brain stimulation studies^22–25^. Using transcranial magnetic stimulations (TMS) over the primary auditory cortex, Kanai *et al*.^24^ observed that time discrimination is impaired not only when auditory signals mark time, but also when visual signals do. However, only the performance in the visual condition is impaired when TMS is used over the primary visual cortex. This finding suggests that in timing tasks the auditory cortex has a supra-modal role: the lower performance in vision than in audition would be due to the need to transfer the visual signals into an auditory code^24^. If such is the case though, having one auditory signal (AV or VA conditions) instead of none (VV condition) should lead once again to performance levels in-between the ones involving two auditory and two visual signals; however, as noted earlier, that is not the case^15,26–28^.

When temporal intervals are marked by different modalities, how does the brain compute time and where in the brain is time represented? Previous studies have led researchers to suggest that, among other regions, the parietal cortex is involved in human time perception in the millisecond-to-second range^29,30^; in particular, some evidence indicates that the parietal cortex is a multimodal region^1,22,31,32^. Indeed, some studies provide support to the idea that multisensory interaction acts in the parietal cortex for goal-directed behaviour to elaborate a dynamic link between sensory stimuli and motor acts^33–37^.

The role of the parietal cortex in temporal processing has been observed in previous fMRI^30,38,39^ and electrophysiological studies^16^, as well as in studies involving non-invasive brain stimulation such as TMS^22,40–42^ and transcranial direct current stimulation (tDCS^43^). In particular, it seems that the right rather than the left parietal cortex is involved in temporal processing of both visual and auditory durations^22,43^.

In the present study, we investigated the effects of inter- and intra-modal stimuli in temporal processing using a non-invasive brain stimulation technique to manipulate the membrane potential of neurons and modulate spontaneous firing rates in the right parietal and in the right frontal cortex. To our knowledge, only two studies on time perception using tDCS^25,43^ have been published so far. In a study involving the reproduction of visual intervals lasting 1500, 1600, 1700, 1800 and 1900 ms, Vicario *et al*.^43^ showed no effect of anodic stimulation (visual stimuli). However, when cathodic stimulation was applied over the right posterior parietal cortex, temporal accuracy was affected, leading participants to overestimate time intervals. Moreover, their results showed that tDCS applied to the left posterior parietal cortex reduced variability when series of temporal intervals were reproduced^43^. In a study by Mioni *et al*.^25^, the authors used a time bisection task in which participants were first trained with two standard durations (standard short = 300 ms and standard long = 900 ms) and then asked to judge whether temporal intervals lasting 300 to 900 ms were closer in duration to the short or to the long standard. Results showed higher variability under anodic stimulations, compared to sham, when the primary auditory cortex was targeted and this result applied when intervals were marked by either visual or auditory signals. Under cathodic stimulation, when the primary visual cortex was stimulated, higher variability was observed only when intervals were marked by visual stimuli.

The temporal tasks used in Mioni *et al*.^25^ (time bisection, sub-second intervals) and Vicario *et al*.^43^ (time reproduction, supra-second intervals) and the different brain areas targeted (primary visual and auditory cortices and posterior parietal cortex) make the comparison of the results from these studies difficult. It is arduous to draw clear conclusions from these studies regarding the different effects of anodic and cathodic stimulation on time perception. More importantly, recent works have been published challenging the anodal-excitation and cathodal-inhibition dichotomy, demonstrating similar effects under both stimulations^44,45^.

In the present study, we opted for a different technique named transcranial random noise stimulation (tRNS), which delivers current at random frequencies. In contrast to tDCS, tRNS has no constraint of current flow direction sensitivity as the intensity and the frequency of the current vary in a random manner. Interestingly, less sensory sensations were reported during tRNS, compared to tDCS^46^. Therefore, the application of tRNS might be better suited for placebo-controlled studies^47,48^. tRNS after-effects are intensity dependent; stimulation at 1.5 mA leading to excitability after-effect is comparable to what has been observed with anodal tDCS, whereas a lower intensity (0.4 mA) leads to inhibitory after-effect comparable with cathodal tDCS^49,50^.

In the present study, we investigated the involvement of right posterior parietal cortex (P4), with tRNS applied in an online procedure, in the processing of inter- and intra-modal temporal intervals. All participants performed the time bisection task under two experimental conditions (random and sham) on two different days. Moreover, we tested a control site (right frontal area, F4) that is not expected to be involved in the processing of brief and multimodal time intervals (<1 sec^51,52^).

With regard to modality effects on time perception, we expected a longer perceived duration (lower bisection point value) and higher sensitivity to time (lower Weber ratio) for conditions involving only auditory rather than only visual signals. More interesting to our research question were the conditions where both auditory and visual stimuli were used to mark intervals (inter-modal conditions). If the auditory vs. visual difference is due to the sensory noise associated with the signals marking an interval, marking an empty interval with one auditory and one visual signal (AV or VA) should lead to performance levels in between the ones involving two auditory (AA) and two visual (VV) signals^2^.

If the parietal cortex is a multimodal region^1,22,32,37^, tRNS on this region should exert greater effect when inter-modal markers (AV, VA), rather than intra-modal markers (AA, VV) are used because of the greater sensory integration needed for switching attention between modalities. Although the physiological mechanisms of tRNS have not been completely clarified yet^47,53^, it has been observed that high-frequency tRNS applied over motor areas (M1) has excitatory effects comparable to what can be observed with anodal tDCS^50,54^. Therefore, if high-frequency tRNS has a facilitatory effect and if the parietal cortex is mainly involved in multisensory integration, it is reasonable to expect an attenuation of the discrepancy between intra/inter-modality temporal performances due to a specific beneficial effect of tRNS in the inter-modal condition.

## Results

**Figure 1:**
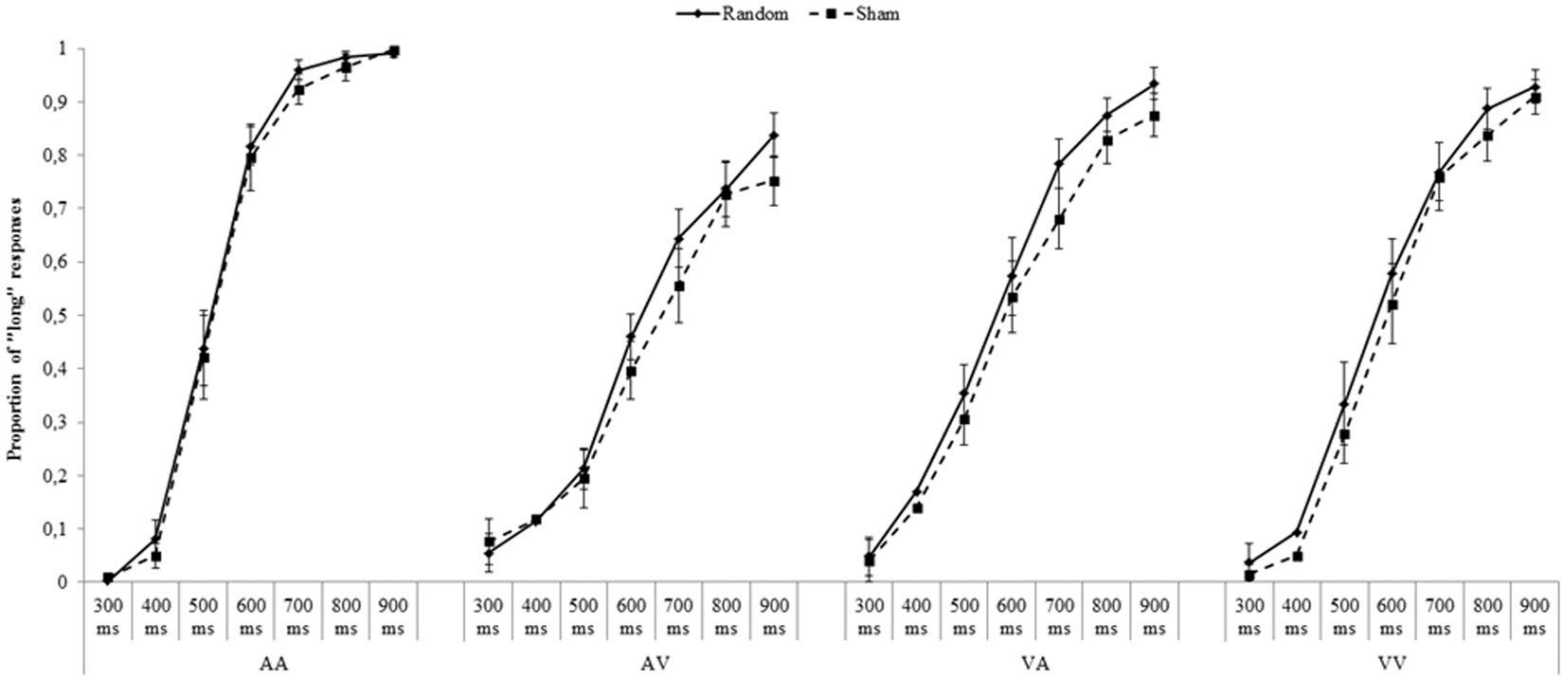
P4: Mean proportion of “long” responses for each *Modality* (AA, AV, VA, VV), as a function of *Stimulation type* (random, sham) and *Temporal intervals* (300–900 ms). Error bars indicate standard errors.

**Figure 2:**
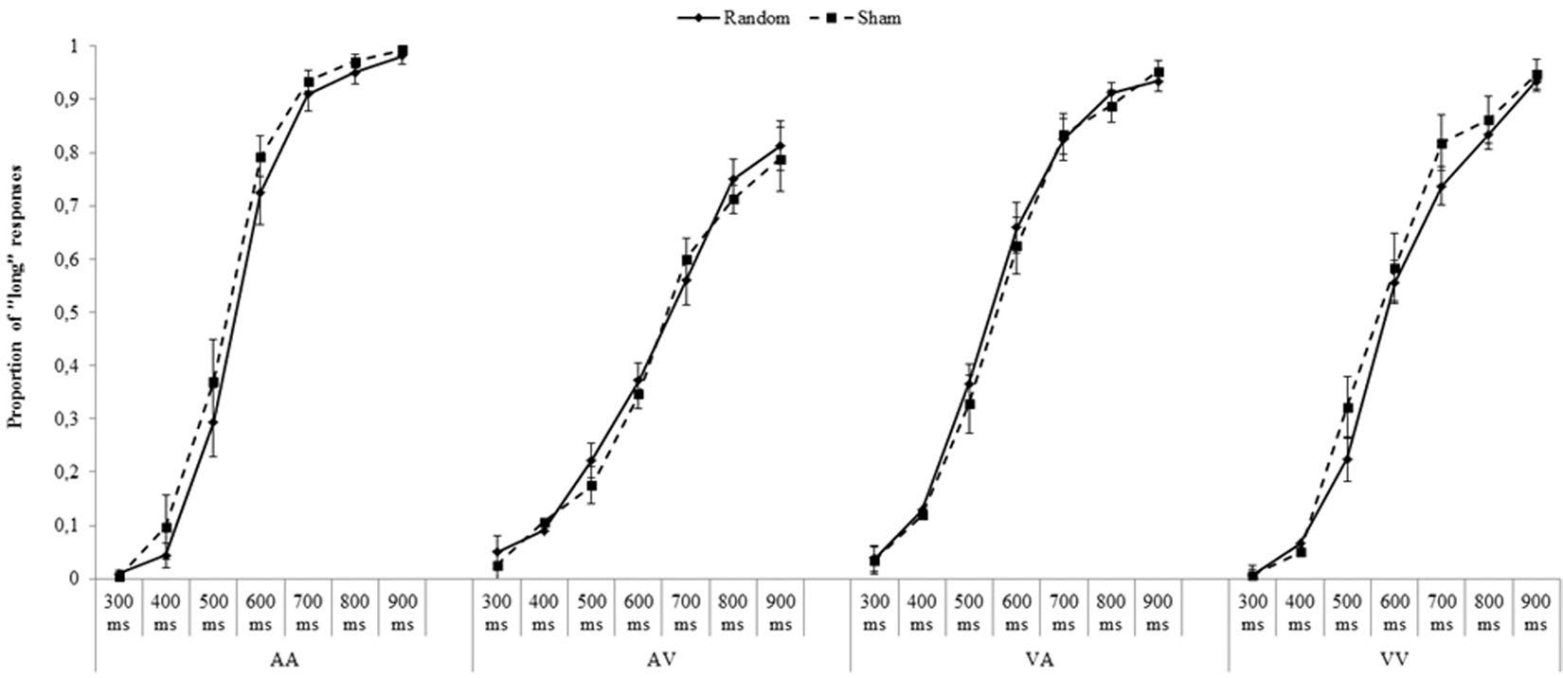
F4: Mean proportion of “long” responses for each *Modality* (AA, AV, VA, VV) as a function of *Stimulation type* (random, sham) and *Temporal intervals* (300–900 ms). Error bars indicate standard errors.

Figures 1 and 2 report the mean proportion of “long” responses for each temporal interval as a function of *Stimulation types* (Random vs. Sham), *Modality* (AA, VV, AV or VA) and both *Areas* (P4 and F4) and Table 1 summarizes the mean PSE in each experimental condition.

**Table 1:** Mean (M) Point of Subjective Equality and standard deviation (SD) in each experimental condition.

		P4	F4
		M (*SD*)	M (*SD*)
Random	AA	524 (47)	558 (53)
	AV	656 (79)	665 (75)
	VA	577 (75)	565 (46)
	VV	591 (88)	618 (37)
Sham	AA	529 (54)	531 (58)
	AV	684 (110)	689 (61)
	VA	614 (91)	574 (50)
	VV	618 (83)	595 (65)

A: Auditory; V: Visual.

**Figure 3:**
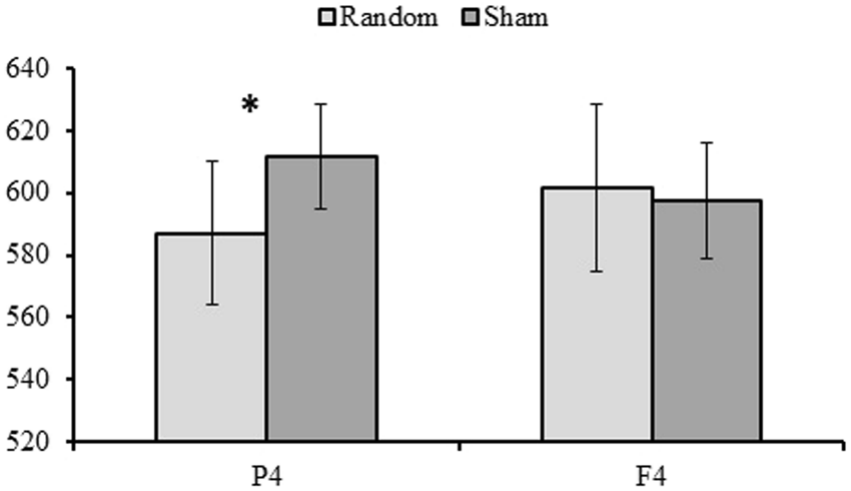
Mean Point of Subjective Equality (PSE) as a function of Area (P4, F4) and Stimulation type (Random, Sham). The error bars indicate standard errors.

**Figure 4:**
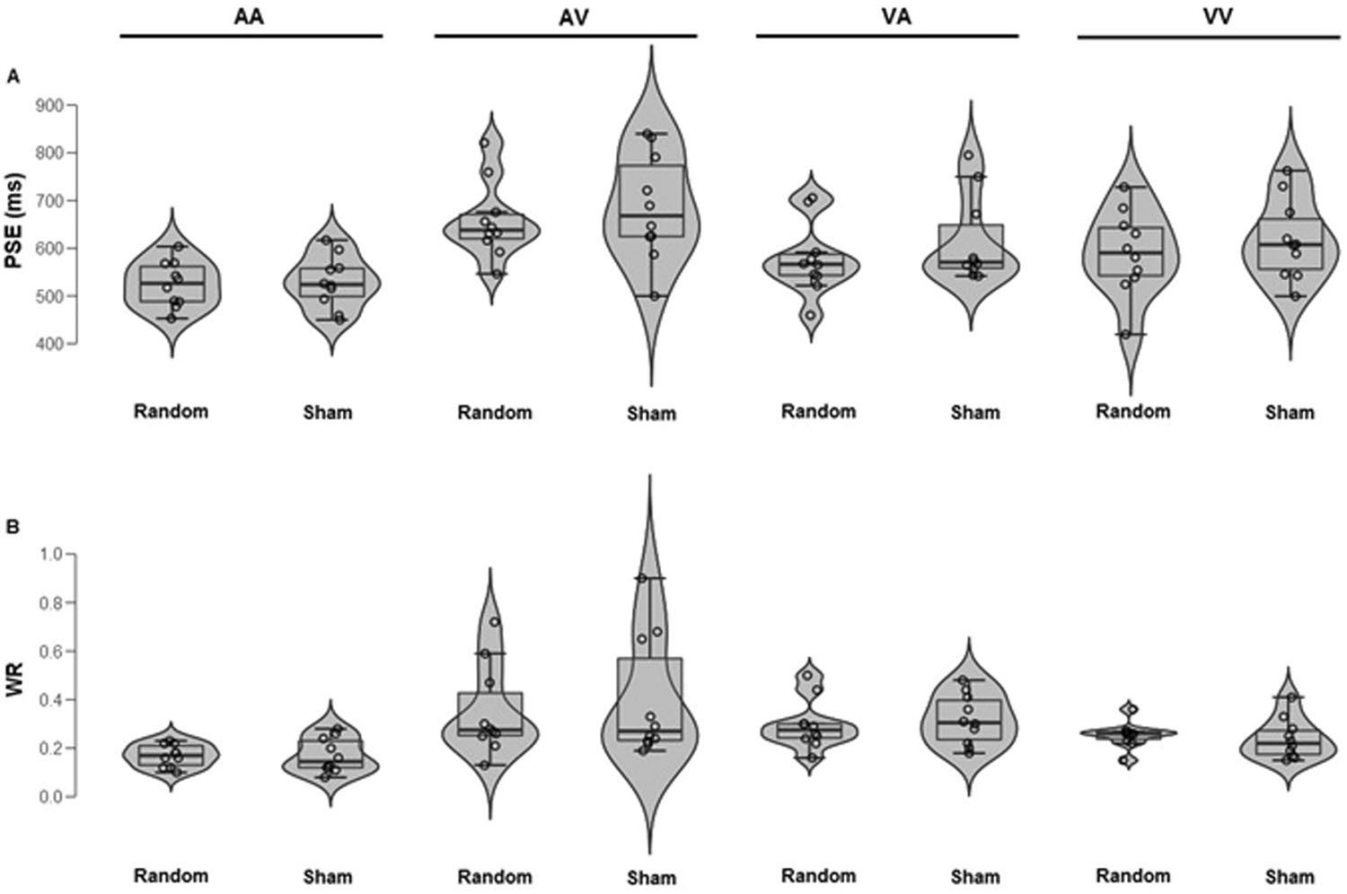
P4: (**A**) Point of Subjective Equality (PSE) and (**B**) Weber ratio (WR) as a function of *Modality* (AA, AV, VA, VV) and *Stimulation type* (random, sham). Each dot represents a participant.

**Figure 5:**
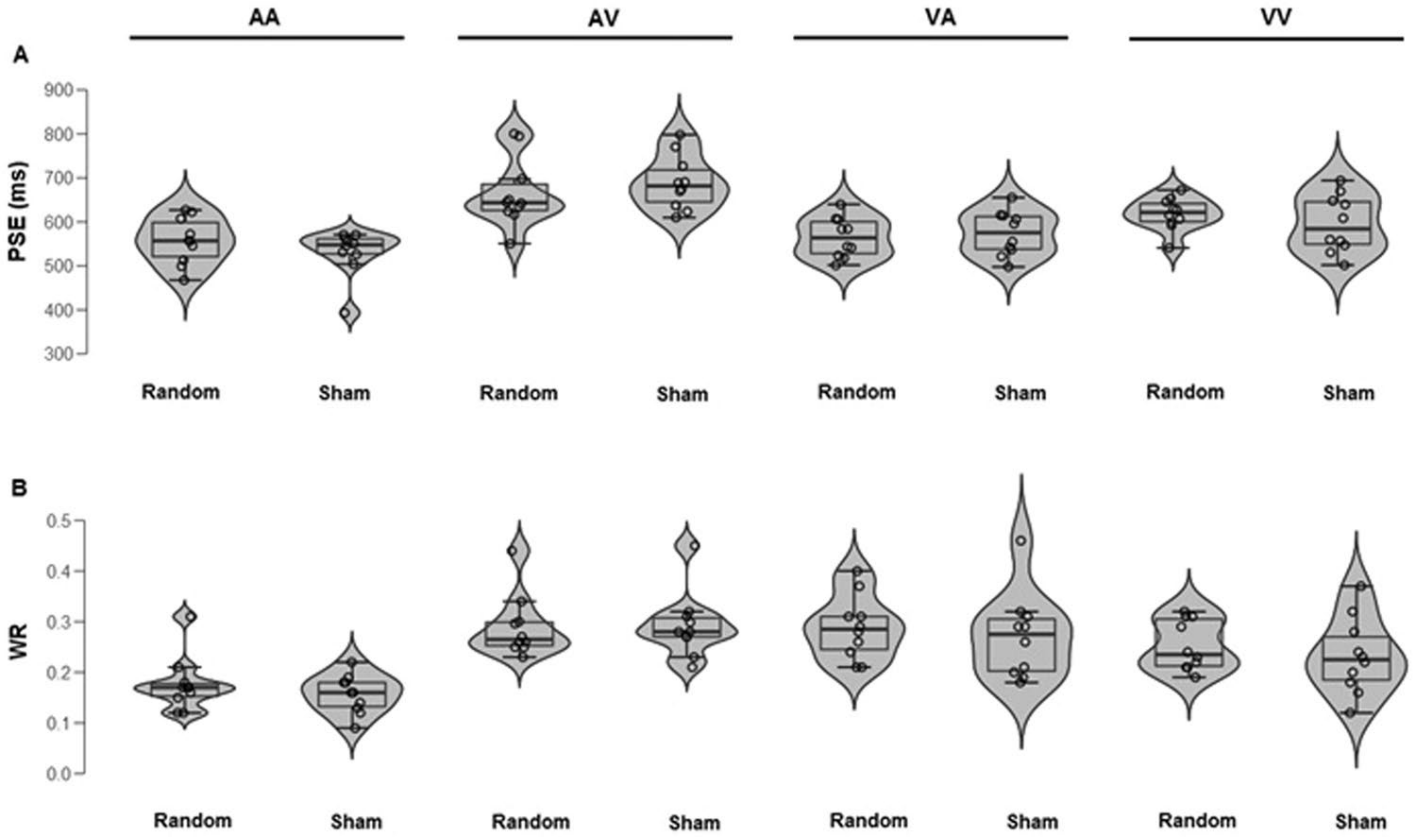
F4. (**A**) Point of Subjective Equality (PSE) and (**B**) Weber ratio (WR) as a function of *Modality* (AA, AV, VA, VV) and *Stimulation type* (random, sham). Each dot represents a participant.

Analyses of PSE showed a significant main effect of *Modality* [*F*(3, 72) = 16.62, *p* < 0.001, η^2^_p_ = 0.41] indicating a higher PSE in the AV modality compared to other modalities (AA = 535 ms, AV = 674 ms, VA = 582 ms and VV = 605 ms). No main effects of *Stimulation type* (*p* = 0.142, η^2^_p_ = 0.03) or *Area* (*p* = 0.987, η^2^_p_ = 0.01) were found but, interestingly, the *Area* × *Stimulation type* interaction was significant [*F*(1, 72) = 4.78, *p* = 0.038, η^2^_p_ = 0.06] (Fig. 3). Post-hoc analyses showed a significant difference between stimulation types when the stimulation was applied over P4 (*p* = 0.013, η^2^_p_ = 0.08), but not when applied over F4 (*p* = 0.657, η^2^_p_ = 0.01). With stimulation over P4, the PSE is lower (longer perceived duration) in the random than in the sham stimulation condition. No other effect was significant (all *p* > 0.322, all η^2^_p_ < 0.01) (Figs 4 and 5, upper parts).

**Figure 6:**
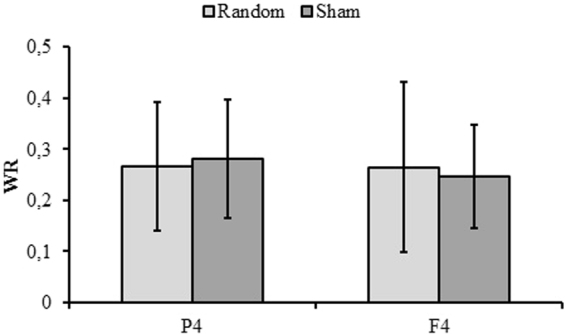
Mean Weber Ratio (WR) as a function of Area (P4, F4) and Stimulation type (Random, Sham). The error bars indicate standard errors.

Analyses of WR showed a significant main effect of *Modality* [*F*(1, 72) = 11.03, *p* < 0.001, η^2^_p_ = 0.31] indicating lower WR in AA (M = 0.17) compared to AV (*M* = 0.35), but no differences involving other modalities were found (VA = 0.29 and VV = 0.24). Neither main effects of *Area* or *Stimulation type* nor significant interactions were found (all *p* > 0.054, η^2^_p_ < 0.05) (Figs 4 and 5, lower parts and Fig. 6).

Analyses of the sensation questionnaire showed no significant main effects of *Modality, Stimulation type, Area* or interaction between these variables (all *p*s ≥ 0.430, η^2^_p_ ≤ 0.04) (Table 2).

**Table 2:** Transcranial random noise stimulation (tRNS)-induced sensations: Mean (M) and standard deviation (SD) intensity of the sensations reported by subjects after tRNS.

Brain area	Stimulation type	Group	Irritation	Pain	Burning	Heat	Itch	Iron taste	Fatigue	M (*SD*)
			M (*SD*)	M (*SD*)	M (*SD*)	M (*SD*)	M (*SD*)	M (*SD*)	M (*SD*)	
P4	Random	AA	0.20 (0.42)	0.20 (0.42)	0.10 (0.32)	0.30 (0.48)	0.20 (0.42)	0.10 (0.32)	0.30 (0.67)	0.20 (0.44)
		AV	0.90 (1.85)	0.20 (0.42)	0.20 (0.63)	0.20 (0.42)	0.30 (0.48)	0.00 (0.00)	0.40 (0.70)	0.31 (0.64)
		VA	0.30 (0.43)	0.10 (0.0.32)	0.10 (0.32)	0.40 (0.52)	0.50 (0.53)	0.00 (0.00)	0.30 (0.48)	0.24 (0.38)
		VV	0.00 (0.00)	0.10 (0.32)	0.00 (0.00)	0.40 (0.70)	0.50 (0.97)	0.00 (0.00)	0.40 (0.70)	0.20 (0.38)
		M (*SD*)	0.35 (1.00)	0.15 (0.36)	0.10 (0.38)	0.32 (0.52)	0.37 (0.62)	0.02 (0.16)	0.35 (0.62)	0.24 (0.52)
	Sham	AA	0.20 (0.42)	0.40 (0.70)	0.20 (0.42)	0.40 (0.70)	0.30 (0.48)	0.10 (0.32)	0.40 (0.70)	0.28 (0.53)
		AV	0.60 (0.84)	0.10 (0.32)	0.50 (0.84)	0.20 (0.42)	0.60 (0.84)	0.10 (0.32)	0.30 (0.67)	0.34 (0.61)
		VA	0.40 (0.70)	0.10 (0.32)	0.10 (0.32)	0.20 (0.42)	0.30 (0.48)	0.00 (0.00)	0.30 (0.42)	0.20 (0.41)
		VV	0.30 (0.67)	0.10 (0.32)	0.1 (0.32)	0.00 (0.00)	0.30 (0.67)	0.00 (0.00)	0.20 (0.42)	0.14 (0.34)
		M (*SD*)	0.37 (0.67)	0.17 (0.45)	0.22 (0.53)	0.20 (0.46)	0.37 (0.63)	0.05 (0.22)	0.30 (0.61)	0.24 (0.51)
F4	Random	AA	0.50 (0.70)	0.20 (0.42)	0.30 (0.67)	0.30 (0.67)	0.50 (0.53)	0.00 (0.00)	0.40 (0.70)	0.31 (0.53)
		AV	0.20 (0.42)	0.00 (0.00)	0.30 (0.48)	0.00 (0.00)	0.10 (0.32)	0.00 (0.00)	0.50 (0.97)	0.15 (0.31)
		VA	0.30 (0.48)	0.00 (0.00)	0.30 (0.67)	0.20 (0.42)	0.50 (0.71)	0.00 (0.00)	0.30 (0.48)	0.23 (0.39)
		VV	0.30 (0.67)	0.00 (0.00)	0.10 (0.32)	0.00 (0.00)	0.20 (0.42)	0.00 (0.00)	0.30 (0.48)	0.13 (0.27)
		M (*SD*)	0.32 (0.57)	0.05 (0.22)	0.25 (0.54)	0.12 (0.40)	0.32 (0.52)	0.00 (0.00)	0.37 (0.67)	0.21 (0.42)
	Sham	AA	0.20 (0.42)	0.10 (0.32)	0.10 (0.32)	0.10 (0.32)	0.20 (0.42)	0.00 (0.00)	0.60 (0.97)	0.18 (0.39)
		AV	0.10 (0.32)	0.00 (0.00)	0.20 (0.42)	0.40 (0.70)	0.40 (0.70)	0.00 (0.00)	0.50 (0.71)	0.23 (0.41)
		VA	0.30 (0.48)	0.00 (0.00)	0.30 (0.48)	0.10 (0.32)	0.50 (0.85)	0.00 (0.00)	0.30 (0.48)	0.21 (0.37)
		VV	0.30 (0.67)	0.00 (0.00)	0.00 (0.00)	0.00 (0.00)	0.30 (0.95)	0.10 (0.32)	0.50 (0. 85)	0.17 (0.40)
		M (*SD*)	0.22 (0.48)	0.02 (0.16)	0.15 (0.36)	0.15 (0.43)	0.35 (0.73)	0.02 (0.16)	0.47 (0.75)	0.20 (0.44)

Sensation intensity was evaluated on a 5-point scale: 0 = none, 1 = mild, 2 = moderate, 3 = considerable, and 4 = strong.

## Discussion

Humans are remarkably proficient at perceiving the passage of time and multiple processes seem to determine subjective perception of current time for intervals lasting several hundredths of milliseconds to several seconds. In particular, differences in temporal processing were observed when temporal intervals were marked by different modalities. These observations are critical in the debate regarding a single “centralized” and “amodal” clock hypothesis vs. an approach promoting multiple timing mechanisms “distributed” across brain areas or circuits^55^. In this second perspective, the engagement of each mechanism depends on the psychophysical task, sensory modality, and lengths of temporal intervals^1,2^. In the present study, for the first time, a high-frequency tRNS was applied over the P4 area to modulate cortical excitability during the temporal processing of intervals marked by auditory and/or visual stimuli using the tRNS technique. The current study uses the biases in temporal perception caused by using inputs from different sensory modalities to specify whether the processing of crossmodal intervals involves a modality-independent mechanism or multiple mechanisms. Our findings provide evidence of the involvement of the P4 area in the representation of time with multimodal stimuli. More specifically, we observed an effect of high-frequency tRNS on P4 independently of the modality used to mark temporal intervals.

The parietal cortex is known as a centre of integration of sensory information and is related to a variety of cognitive functions^29,56^. Its anatomic-functional relations with the temporal and dorsolateral prefrontal cortex are also associated with action control and spatial reference. In this context, parietal cortex is essential in planning movements based on sensory information and in coding cognitive functions. Thus, the perception of external stimuli is integrated by the parietal cortex to the timing of intervals lasting a few milliseconds up to a few seconds^57^.

No effect of stimulation on perceived duration (PSE) or sensitivity (WR) was observed when high-frequency tRNS was applied over F4. These results are in line with our prediction of limited involvement of frontal areas in temporal processing when brief temporal intervals are processed^51,58,59^. It has been suggested by Lewis and Miall^51^ that time processing in the shorter range (approximately below 1 sec) is ‘automatic’, reflecting the engagement of processes associated with the production of skilled movements. Longer time range is hypothesized to be ‘cognitive’, dependent on neural systems associated with attention and working memory^52,60^.

Regarding the specific effect of tRNS on time processing, differently from previous studies conducted with TMS^22,24^ and with tDCS^25^, we did not observe any effect of stimulation on temporal sensitivity. Compared to the sham condition, participants under random stimulations over P4 rather overestimated time. This effect on perceived duration did not affect temporal sensitivity.

Fewer studies have been conducted with tRNS than with tDCS and, therefore, the behavioural and physiological mechanisms underlying tRNS effects are not yet completely understood. At the behavioural level, Fertonani *et al*.^61^ showed a significant enhancement in visual perceptual learning during the online application of high-frequency tRNS over visual cortices, and Romanska *et al*.^62^ showed that tRNS over the lateral occipital cortex facilitated facial identity perception. In contrast, tRNS applied to the right dorsolateral prefrontal cortex (DLPFC) impaired categorical learning in a prototype distortion task^63^. These results demonstrate that depending on the cortical area involved and the type of protocols, tRNS can induce long-term positive but also negative changes in cognitive and brain functions. Moreover, no effect of tRNS over the DLPFC on working memory performance was observed^64^.

One potential effect of tRNS might be related to the improvement of the signal-to-noise ratio in the central nervous system and to the sensitization of sensory processing^53,65^. It was suggested that tRNS may increase synchronization of neural firing through amplification of sub-threshold oscillatory activity, which in turn reduces the amount of endogenous noise^65^. The effects of tRNS might also be associated with repetitive opening of Na^+^ channels, as it was observed in a study investigating the application of alternating current stimulation to rat hippocampal slices^66^. Finally, it is proposed that tRNS might induce long-term hemodynamic changes in the human brain that could be related to brain plasticity reorganization^67^. Besides, the effects of tRNS might be based on other mechanisms, such as stochastic resonance^68^. Briefly, stochastic resonance refers to the phenomenon that a signal too weak to exceed a threshold is amplified by adding noise, for example, when a neural oscillation in the brain is sub-threshold. These probably synaptically operated sub-threshold activities, driven by oscillatory inputs that neurons receive from other brain regions, are not strong enough to induce action potential generation. If random noise is added, the sum of the two signals exceeds the threshold at certain times. The frequency of the supra-threshold signal is determined by the existing sub-threshold neural oscillation. It has been suggested that tRNS may increase synchronization of neural firing through amplification of sub-threshold oscillatory activity, which in turn reduces the amount of endogenous noise. The improvement of the signal-to-noise ratio in the central nervous system and the sensitization of sensory processing can lead to enhanced perception or cognitive performance^65,69^.

As regards the performance levels observed in the present study, the WR in different conditions are a little high, at least in AA (0.17) and VV (0.24); in other reports, these values are much lower in VV^13,70^. One potential explanation is the large distribution of interval values used to build the psychometric functions in this study. This probably led to an underestimation of participants’ capabilities. Grondin^71^ reported that the distribution of interval values (and the number of intervals used to build the psychometric functions) has a negligible impact on the estimation of performance. However, the distribution used in the present study (300 to 900 ms for a mid-point at 600 ms) is much larger than Grondin’s^71^ in the largest-value conditions; moreover, in Grondin’s^71^ work the investigation was limited to the AA condition. The possibility that the large distribution of interval values has led to an underestimation of AA and VV performances may well have contributed to decreasing the difference most often observed when performances in intra- and inter-modal conditions are compared. For brief intervals, discrimination is usually better in VV than in AV or VA^15,16,27,72^, but the differences were not found to be significant in the present study. In any case, the present results replicate the superiority of the AA condition for temporal processing over modality conditions.

Limitations of the present study are the sample size and the unequal number of males and females included. While acknowledging that the groups are small, we believe that our study can provide interesting insights into the understanding of the brain areas and networks involved in temporal processing despite the localization inaccuracy inherent to the tRNS.

As far as we know, no previous studies have been conducted using tRNS for investigating the mechanisms involved in the processing of temporal information. The effect size in our previous study (Mioni *et al*.^25^), where tDCS was applied over A1 and V1, was around 0.15, which is admittedly small. Future studies should further analyse the specific effect of tRNS on perceived duration (PSE) and not only on temporal variability (WR). What is the source of this effect? Direct comparison between our and previous studies are difficult considering the different areas targeted. It is possible that different structures respond differently to continuous or random stimulation or it is possible that the specificity of each stimulation, promoting different excitatory effects, acts either on sensitivity or on perceived duration?

In brief, the study shows that perceived duration is changed by tRNS when applied to P4, but not when applied to F4. In the context of a multi-modal investigation, this finding is interesting because the P4 area is associated with sensory processing, while F4 is not.

## Method

### Procedure

Participants were tested in two different sessions (random or sham stimulation) on two different days counterbalanced between participants. Between sessions, there were at least 48 h to avoid long lasting effects of the stimulation^73^. Half of the participants were randomly assigned to one of the two stimulated area (P4 or F4). Within each condition, 10 participants were randomly assigned to one of the four modality conditions: AA, AV, VA, or VV. During each session, participants first performed the time bisection task and then the sensation experienced questionnaire to control for possible inconveniences induced by the stimulation. Instructions and training were conducted off-stimulation and the stimulation started after the training phase. This procedure was adopted to avoid any effect of stimulation during the training phase^25^.

### Participants

Eighty participants were tested and recruited at the Department of General Psychology, University of Padova (Italy). Forty University students were stimulated over P4 (mean age = 23.04, *SD* = 1.87; Male = 20) and 40 over F4 (mean age = 22.88 years, *SD* = 2.45; Male = 10). All participants were right-handed according to the Edinburgh Handedness Inventory scores^74^. The study was approved by the Ethics Committee of the Department of General Psychology of Padova (Italy) and conducted according to the Declaration of Helsinki (59^th^ WMA General Assembly, Seoul, 2008). All participants gave their informed written consent before participating in the study. Exclusion criteria included a history of neurological or psychiatric illness, pregnancy, and use of drugs or alcohol 24 h prior the experimental sessions. Participants were informed about the objective of the study only after completing the second experimental session.

### Time bisection task

Each session started with the learning phase, in which participants were required to memorise the two standard durations: 300 ms (short standard) and 900 ms (long standard). Both standard durations were presented 10 times in a fixed order (300–900 ms). After the learning phase, participants were required to judge different temporal intervals (testing phase: 300, 400, 500, 600, 700, 800, 900 ms) and decide if the comparison interval was more similar to the short standard or to the long standard. We used the “A” and “L” letters of the QWERTY keyboard, on which we used the label “B” and “L”, or “L” and “B”, during the task. Participants were required to press the key labelled “B” (“B” refers to the Italian word “Breve” = short), if the duration presented was closer to the short standard, or to press the key labelled with “L” (“L” refers to the Italian word “Lungo” = long), if the duration presented was closer to the long standard. In the present study we used empty intervals marked by two signals of the same modality (intramodal intervals: AA or VV) or by two signals from different modalities (intermodal intervals: AV or VA). The sensory signals used to mark the two standard durations learned by participants were the same as the ones used in the testing phase. The visual marker was a black dot (1 cm diameter) presented at the centre of the computer screen for 50 ms, while the auditory marker was a white noise presented for 50 ms through speakers. Participants were seated 60 cm from the computer screen. Each comparison duration was presented 8 times for a total of 56 trials in each block; participants performed 4 blocks for a total of 224 trials. The participants were asked to respond with their left and right index finger and response keys were counterbalanced between participants. After the response, there was a 1000-ms inter-trial interval.

### tRNS stimulation

High-frequency tRNS was delivered using a battery-driven stimulator (BrainSTIM, EMS) through a pair of saline-soaked sponge electrodes. The tRNS consisted of an alternating current of 1.5 mA intensity with a 0-mA offset applied at random frequencies. The frequencies ranged from 100 to 640 Hz. The stimulation was applied for approximately 10 min during the testing phase, but not during the learning phase. This procedure was adopted in order to avoid the effect of stimulation on the two standard intervals^25^. The active electrode had an area of 16 cm^2^ and the reference electrode had an area of 60 cm^25,75^. The current density was maintained well below the safety limits and the electrodes were kept in place with bandages. The stimulating electrode was placed over P4 or F4 according to the international 10/20 system for EEG electrode. We decided to use an extra-cephalic montage for the reference electrode that was placed over the right shoulder. The choice of an extracephalic montage was to avoid any confounding effect in the brain that could derive from the positioning of the reference electrode and the same montage was successfully used in a previous study on time perception using tDCS^25,43^. As far as we know this is the first time that this montage is used with tRNS in the field of temporal processing. The sham stimulation consisted of the first 30 s of real stimulation in order to give participants the sensation of electrical stimulation. Even in this case, the electrode was placed over P4 or F4 regions. All participants included in the study completed all sessions.

### Sensation experienced questionnaire

We included a questionnaire about the sensations experienced during the different stimulations (random or sham^76^). The questionnaire includes 8 possible sensations commonly experiences during stimulation. Participants were asked to rate each intensity on a Likert scale from “0 = not experienced” to “4 = intense sensation”. The questionnaire was introduced to evaluate whether unspecific stimulation effects related to different conditions could account for differences in behavioural performance.

### Data analyses

For each participant in each experimental condition, a 7-point psychometric function was traced, plotting the seven comparison intervals on the *x*-axis and the probability of responding “long” on the *y*-axis. The cumulative normal function was fitted to the resulting curves. More specifically, we used a non-linear least squares analysis, with a Levenberg-Marquardt algorithm. For each condition of each participant, the goodness-of-fit was highly satisfactory, with R^2^ values above 0.80 for the random and above 0.81 for the sham conditions; and the Kolmogorov-Smirnov test showed that all the variables were normally distributed.

To further explore the effect of stimulation on brain areas and modality condition, we calculated two indexes, one that defines pe We decided to use an extra rceived duration and one for sensitivity. The first was the Point of Subjective Equality (PSE), that is, the stimulus duration at which the participants responded “short” or “long” with equal frequency. An observed shift of the PSE can be interpreted as an indicator of differences in perceived duration, with smaller PSE values meaning longer perceived durations. The second dependent variable was the Weber ratio (WR), which is based on one standard deviation (SD) of the psychometric function and is an index of time sensitivity. The WR is the SD divided by 600 ms, which is the midpoint duration used in the experiment^77^. Data were analysed in terms of PSE and WR as dependent variables, conducting a repeated measure ANOVA with *Area* (P4 and F4) and *Modality* (AA, AV, VA, or VV), as between-subjects factors and *Stimulation type* (random vs. sham) as within-subjects factor. Data from the sensation experienced questionnaire were analysed adding the values participants reported for a specific sensation at the end of each session. We calculated a total index of sensation for random or sham sessions. A repeated measure ANOVA was conducted and the factors were as for the previous analyses.

The significant analyses were followed by post-hoc analyses with Bonferroni’s correction, to reduce the Type I error rate, and the effect size was estimated with the partial eta squared index.

### Data availability

The datasets generated during and/or analysed during the current study are available from the corresponding author on reasonable request.
